# Glasgow Royal Infirmary and the Age Limit

**Published:** 1906-02-17

**Authors:** 


### Glasgow Royal Infirmary and the Age Limit.

The qualified contributors to the Glasgow Royal Infirmary were provided at their recent annual meeting with a very unusual experience. This appeared in the shape of a proposal, the object of which was, in essence, to permit certain members of the medical staff to evade one of the rules of the hospital. Under the rule in question—it is a frequent regulation in hospital organisations—a physician or surgeon is compelled to retire from the charge of wards on reaching the age of 60 years. Three members of the Royal Infirmary staff have recently attained to this position. Yet the claim is advanced, not that the rule should be cancelled, but that while remaining in operation against other members of the staff it should at the present juncture be suspended in order to avoid the sacrifice of the sacred three. Such a demand, it will be allowed, is a very extraordinary one, and those to whom it is addressed may reasonably claim to hear from its sponsors some substantial justification of their peculiar request. So far as the matter has gone at present it cannot be said that this claim has been satisfied, and on the facts that have reached us we are of opinion that the managers will be well-advised to adhere to the deliberate and well-considered policy of the hospital. That policy is supported by the custom prevalent in the majority of the other large hospitals of the country, and by the practice adopted in most branches of the public service. Further, it has in the case of former members of the staff of the Glasgow Royal Infirmary been accepted without question or complaint. Why, then, it may be asked, should it be challenged on the present occasion? The only argument that has been made public is that the three members of the staff who now fall to retire under the age limit are also teachers in St. Mungo's College, this being the titular distinction of the medical school associated with the Royal Infirmary. The suggestion, presumably, is, that if these gentlemen are deprived of their wards their teaching in the College will be less efficient, and that the interests of the College, and indirectly also those of the hospital, will be prejudiced. That may possibly be a question for the governors of the College, whose duty it is to see that inefficiency in the teaching is prevented by a scheme of retirement that will secure new teachers as these become necessary; but it affords no reason why the managers of the hospital should continue certain members of their staff in office in defiance of the rules of the institution. The claim made in the supposed interests of the College becomes still less substantial when it is discovered that of the three gentlemen with whose positions we are now concerned two are not systematic lecturers in the school, but merely clinical teachers in the hospital wards. The teaching work they undertake, therefore, differs in no respect from that performed by other members of the hospital staff. Hence their retiral from the wards has no more penalty for the hospital and school than has the retiral of any other of the physicians and surgeons. It is an incident not free from pain and regret, but it occurs in accordance with a rule widely recognised as of public value and importance. The motive of that rule is not merely, or even mainly, that age brings limitations to all men. Rather it rests on a recognition of the fact that to attract the best brains and energies to the hard work and postponed rewards of hospital service it is essential that duty well performed in the subordinate posts shall carry the certainty of promotion to larger opportunities. Remove this prospect, which depends on the regular occurrence of vacancies in the senior appointments, and talent and energy will find other fields. It is these large and non-personal considerations which must guide the managers of our hospitals. We urge them on the present occasion in opposition to a proposal which, by prejudicing the reasonable ambition of the younger men and establishing inequitable distinctions between the senior members of the staff, is contrary to the best and most enlightened traditions of hospital management.

